# Subcortical tau burden correlates with regional brain atrophy and plasma markers in four-repeat tauopathy parkinsonism

**DOI:** 10.1177/1877718X241298192

**Published:** 2024-12-08

**Authors:** Cheng-Hsuan Li, Sung-Pin Fan, Ming-Chieh Shih, Yi-Hsin Weng, Ta-Fu Chen, Hsun Li, Mei-Fang Cheng, Ming-Che Kuo, Pei-Ling Peng, Makoto Higuchi, Ing-Tsung Hsiao, Kun-Ju Lin, Chin-Hsien Lin

**Affiliations:** 1 Department of Neurology, National Taiwan University Hospital, Taipei, Taiwan; 2 Department of Neurology, National Taiwan University Hospital Hsinchu Branch, Hsinchu, Taiwan; 3 Graduate Institute of Clinical Medicine, College of Medicine, National Taiwan University, Taipei, Taiwan; 4 School of Medicine, College of Life Sciences and Medicine, National Tsing Hua University, Hsinchu, Taiwan; 5 Department of Neurology, Linkou Chang Gung Memorial Hospital, Taoyuan, Taiwan; 6 Neuroscience Research Center, Linkou Chang Gung Memorial Hospital, Taoyuan, Taiwan; 7 Department of Nuclear Medicine, National Taiwan University Hospital, Taipei, Taiwan; 8 Department of Medicine, National Taiwan University Cancer Center, Taipei, Taiwan; 9 Institute for Quantum Medical Science, Quantum Life and Medical Science Directorate, National Institutes for Quantum Science and Technology, Chiba, Japan; 10 Nuclear Medicine and Center for Advanced Molecular Imaging and Translation, Linkou Chang Gung Memorial Hospital, Taoyuan, Taiwan; 11 Department of Medical Imaging and Radiological Sciences, Chang Gung University, Taoyuan, Taiwan; 12 College of Medicine, National Taiwan University, Taipei, Taiwan; 13 Graduate Institute of Biomedical Engineering, College of Medical Science and Technology, Taipei Medical University, Taipei, Taiwan; 14 Graduate Institute of Molecular Medicine, College of Medicine, National Taiwan University, Taipei, Taiwan; 15 Graduate Institute of Brain and Mind Sciences, College of Medicine, National Taiwan University, Taipei, Taiwan

**Keywords:** alzheimer's disease, corticobasal syndrome, ^18^F-Florzolotau, parkinsonism syndrome, progressive supranuclear palsy, tau

## Abstract

**Background:**

^18^F-florzolotau positron emission tomography (PET) assists in the *in vivo* diagnosis of progressive supranuclear palsy (PSP).

**Objective:**

We aimed to investigate the relationship between ^18^F-florzolotau uptake and clinical severity, structural volume changes, and plasma markers in four-repeat tauopathies.

**Methods:**

A total of 80 participants were recruited: 35 with PSP (11 with PSP-Richardson syndrome and 24 with PSP non-Richardson syndrome), 9 with corticobasal syndrome (CBS), 10 with Alzheimer's disease (AD), 8 with Parkinson's disease, and 18 controls. All participants underwent ^18^F-florzolotau PET, brain magnetic resonance imaging (MRI), and plasma biomarker investigation (total and phosphorylated tau [pTau181], neurofilament light chain, and glial fibrillary acidic protein [GFAP]).

**Results:**

^18^F-Florzolotau uptake was significantly higher in the subcortical regions of the pallidum, subthalamic nucleus (STN), midbrain, red nucleus, and raphe nucleus in PSP patients compared to the other groups (all *p *< 0.01). Subcortical tau tracer retention assisted in distinguishing PSP and CBS from controls (AUC = 0.836, *p *< 0.001). Tau tracer retention could differentiate PSP and CBS from AD in cortical (*p *< 0.001) and subcortical regions (*p *= 0.028). The motor severity of PSP positively correlated with tau burden in STN (*p *= 0.044) and substantia nigra (*p *= 0.035). Tau tracer uptake was associated with cortical volume changes in CBS (*p *= 0.031), PSP non-Richardson syndrome (*p *= 0.003), and AD (*p *= 0.044). Cortical tau retention correlated with plasma levels of GFAP (*p *= 0.001) and pTau181 (*p *= 0.036).

**Conclusions:**

Subcortical ^18^F-Florzolotau uptake assist the diagnosis of 4R tauopathy parkinsonism. Additionally, regional tau burden contributes to structural brain volume changes and correlates with plasma levels of GFAP and pTau181.

## Introduction

Atypical parkinsonian syndromes consist of a heterogeneous group of neurodegenerative disorders, including progressive supranuclear palsy (PSP), corticobasal syndrome (CBS), and multiple system atrophy (MSA). Patients with atypical parkinsonian syndromes are characterized by a more rapid deterioration and poorer levodopa response than those with Parkinson's disease (PD).^1^ Among these syndromes, PSP and CBS are primary 4-repeat (4R) tauopathy disorders, constituting the most common tauopathy after Alzheimer's disease (AD), which has mixed 3R and 4R tau deposition.^2^ Unlike PD, there are no effective treatments for PSP and CBS, and a high degree of clinical overlap between these 4R tauopathy parkinsonism syndromes and PD causes tremendous difficulties in early diagnosis and treatment.^3^ Furthermore, aside from the classical PSP-Richardson syndrome (PSP-RS) phenotype,^4^ the phenotypic spectrum of PSP includes variable nonRS clinical subtypes.^5^ These different subtypes of PSP make early and accurate diagnosis more challenging. In addition, the pathological heterogeneity of CBS encompasses diverse diseases, such as the 4R tauopathy of corticobasal degeneration, mixed 3R and 4R tauopathy of AD, and frontotemporal degeneration with TDP-43.^6^ These heterogeneous clinical and neuropathological features necessitate sensitive and quantitative markers for investigating tau deposition in patients with 4R tauopathy parkinsonism syndrome.

The advent of tau-targeting therapies focusing on multiple aspects of tau pathology, including reducing tau expression utilizing anti-sense oligonucleotides, modulating post-translational modifications, inhibition of tau aggregation, stabilization of microtubules and immnotherapeutics, have entered clinical trials in patients with tauopathy disorders, including PSP.^7,8^ However, though one recent clinical trial reported safe reduction of tau levels in the cerebrospinal fluid (CSF) of participants below baseline, clinical symptoms were not significantly different between the placebo and treatment groups.^9^ Identifying surrogate markers to recognize patients with evidence of tau pathologies early could allow anti-tau therapies to be tailored to mitigate disease progression.

Recently, the second-generation tau tracer for positron emission tomography (PET), ^18^F-florzolotau (also known as ^18^F-APN-1607 or ^18^F-PM-PBB3) was demonstrated to bind 4R tau and assisted in pre-mortem detection of pathological tau depositions in patients with PSP and CBS.^10,11^ However, ^18^F-florzolotau uptake was also increased in the basal ganglia and dentate nucleus of patients with MSA, which is an α-synucleinopathy rather than tauopathy.^12^ These observations highlight the need for multifaceted tau-directing markers to assist in early identification of patients with atypical parkinsonism. In addition to tau PET imaging, CSF concentrations of phosphorylated tau and neurofilament light chain (NfL) have been shown to correlate with disease severity and progression of PSP.^13^ The relatively invasive procedure for collecting CSF necessitates investigating serum or plasma as alternatives. Here, we examined the ability of multimodal tau-directing imaging and biofluid markers to differentiate 4R tauopathy parkinsonism syndrome from healthy controls and other disease groups. We also investigated the regional tau tracer uptake in ^18^F-florzolotau PET scans and the relationship with clinical severity, structural brain imaging, and plasma markers in patients with 4R tauopathy parkinsonism syndrome.

## Methods

### Participants

A total of 80 participants with PSP-RS subtype (n = 11), PSP-nonRS subtype (n = 24), CBS (n = 9), AD (n = 10), PD (n = 8) and neurologically normal controls (n = 18) were recruited from movement disorder clinics in National Taiwan University Hospital between January 2019 and December 2022. Patients were diagnosed according to clinical diagnostic criteria or guidelines for PSP, CBS, PD, or AD.^14–17^ Subtypes of PSP were identified and designated as PSP-RS subtype or PSP-nonRS subtype in accordance with the Movement Disorder Society diagnostic criteria.^14^ CBS was diagnosed based on Armstrong criteria.^15^ Patients with AD met the 2011 National Institute on Aging and Alzheimer's Association (NIA-AA) diagnostic criteria.^17^ Control participants, who were accompanying friends or spouses of the patients, were older than 40 years of age with no history of neurological or psychiatric disorders and had a Mini-Mental Status Examination (MMSE) score ≥ 28. Patients with a history of traumatic brain injury, brain surgery, stroke, or major medical illnesses were excluded. The study protocol complied with the tenets of the Declaration of Helsinki and was approved by the research ethics committees from National Taiwan University Hospital (IRB: 202006181MINB). Written informed consent was obtained from all participants and legal guardians.

### Clinical assessment

All patients with PSP, CBS, or PD were assessed at least 8 h after their last dose, and most had discontinued their medication for 12 h to achieve the trough level of the last anti-parkinsonian medication. For motor symptom severity, patients with PD or CBS were assessed by the Movement Disorder Society Unified Parkinson's Disease Rating Scale (MDS-UPDRS) part III during the off status.^18^ The motor severity of PSP was assessed by the PSP Rating Scale for patients off medication.^19^ Cognitive function was assessed by the MMSE^20^ and Montreal Cognitive Assessment (MoCA).^21^ The total levodopa equivalent daily dose was calculated for patients with PSP, CBS, and PD.

### Image acquisition

The tracer, ^18^F-florzolotau, was prepared and synthesized at the cyclotron facility of Chang Gung Memorial Hospital and sent to the Center for Nuclear Imaging of National Taiwan University Hospital. All participants underwent a 20 min ^18^F-florzolotau PET scan 90 min after injection of 189 ± 27 MBq. All PET scans were performed in a Biograph mCT PET/CT system (Siemens Medical Solutions, Malvern, PA) or a Discovery 710 PET/CT system (GE Medical Systems, Milwaukee, MI). For the mCT PET/CT system, a 3D OSEM algorithm was applied with the following reconstruction parameters and CT-based attenuation, scatter, and random correction: 4 iterations, 24 subsets, and 2 mm FWHM Gaussian filtering (zoom = 3) for post-smoothing. The resulting images had a matrix size of 400 × 400 × 111 and voxel size of 1.02 × 1.02 × 2.03 mm^3^. For the Discovery 710 PET/CT system, images were reconstructed using a 3D OSEM algorithm of 3 iterations, 16 subsets, and 5 mm FWHM post-smoothing with a matrix size of 256 × 256 × 47 and voxel size 1.56 × 1.56 × 3.27 mm^3^. The PET images were motion-corrected and averaged into 20 min static for later processing. The tau PET images were pre-processed using pre-optimized scanner-specific filters from a previous phantom study, so that all images from different scanners resulted in unified resolution for further analysis.^22^

Each participant underwent T1-weighted magnetic resonance imaging (MRI) on a 3-T Siemens Magnetom TIM Trio scanner (Siemens Medical Solutions, Malvern, PA) for delineation of volumes of interest (VOIs) and spatial normalization. Whole brain high-resolution T1-weighted images were acquired using the following parameters: echo time, 2.43–4.65 milliseconds; repetition time, 1600–2670 milliseconds; flip angles, 8–12°; 170 slices; slice thickness, 1 mm; FOV, 224 mm; image matrix, 224 × 224; voxel dimensions, 0.5 × 0.5 × 1 = 0.25 mm^3^.

### ^18^F-Florzolotau PET image processing

The multimodality image processing software Pmod (version 4.2, PMOD Technologies Ltd, Zurich, Switzerland) was applied for the quantitative image analysis. Individual ^18^F-florzolotau PET scans were resampled in the common space of the corresponding T1-weighted images and subsequently registered to the Montreal Neurological Institute (MNI) brain template as described previously.^22^ The regional standard uptake value ratio (SUVR) for VOIs, including individual cortical regions and subcortical regions of the amygdala, putamen, pallidum, thalamus, subthalamic nucleus (STN), red nucleus, substantia nigra, raphe nucleus, midbrain, and dentate nucleus, were generated and modified from the automated anatomic labeling (AAL) atlas. The cortical regions were mainly consisted of gray matter voxels. The inferior cerebellar cortex (crus) was applied as the reference region for calculating the regional SUVR. In addition, a global cortical SUVR was calculated by taking the mean SUVR within the cortex area, including the temporal, frontal, parietal, and occipital regions, and a global subcortical SUVR was measured by taking the mean SUVR from the aforementioned subcortical regions.

### MRI volumetry analysis

Structural MR images were pre-processed with the re-aligned structural high-resolution T1 images using the automated segment pipeline of Computational Anatomical Toolbox 12 (CAT12) in Statistical Parametrical Mapping 12 (SPM 12). The AAL atlas was applied for VOI processing. Total cortical gray matter (GM) volumes were also calculated in CAT12, which were mainly gray matter voxels. The subcortical total GM volumes and regional volumes were estimated using the Brainstem Substructures Tools and the original output in FreeSurfer (version 7.1.0, http://surfer.nmr.mgh.harvard.edu/). The left and right volumes of the above regions were averaged and divided by the individual estimated intracranial volume (eTIV), representing regional to total volume ratios.

### Plasma biomarkers

Samples of 10 mL venous blood were collected at the time of enrollment and centrifuged (2500 g for 15 min) within 1 h of collection. The plasma aliquots were stored at −80°C until testing. Plasma levels of total tau, phosphorylated tau-181 (pTau181), NfL, and glial fibrillary acidic protein (GFAP) were quantified using a single molecule array (Simoa; Quanterix, Billerica, MA, USA). All samples were run in duplicate. Measurements were performed by a laboratory technician who was blinded to the clinical diagnosis.

### Statistical analysis

All continuous variables are presented as means and standard deviations (SDs), and nominal variables as numbers and percentages. Baseline characteristics between different groups were compared by analysis of variance (ANOVA) or the Kruskal–Wallis test when appropriate; chi-squared test was used for nominal data. The Shapiro–Wilk test and Levene's test were used for testing assumptions of normal distribution and equality of variances. For tau deposition assessed by ^18^F-florzolotau, we used analysis of covariance (ANCOVA) to compare the SUVR in each VOI between different disease groups after adjusting for age and sex. Bonferroni correction was used for post hoc between-group comparisons. Receiver operating characteristics (ROC) analysis was performed for differentiation of 4R tauopathy parkinsonism syndrome (PSP and CBS) from control or AD groups based on the cortical or subcortical tau load or gray matter structural volume ratio. For the structural volume from MRI, the volume ratios between disease groups were compared by ANCOVA with post hoc Bonferroni correction adjusted for age and sex.

The relationship between tau PET and MRI volume was explored using the partial least squares (PLS) method.^23^ There are several advantages to using the PLS method to test the relationship between tau PET and brain volume. First, both the ^18^F-Florzolotau SUVR and volume ratios are multivariate signals (one signal per VOI). PLS provides a robust approach to assess the overall correlation between these multivariate signals without the need for multiple comparison adjustments. Second, PLS is commonly used in neuroimaging studies and has well-established routines.^23,24^ Additionally, PLS generates VOI patterns that reveal where the correlation between SUVR and volume is strongest, which enhances interpretability and helps identify plausible biological mechanisms. Briefly, the z-transformation of SUVR and volumes was performed based on the entire sample, including controls, in accordance with standard procedures for PLS analysis. The Z-transformation of the ^18^F-Florzolotau SUVR and volume ratios in multiple selected VOIs (frontal, temporal, parietal, occipital, amygdala, putamen, pallidum, STN, midbrain, substantia nigra, red nucleus, and raphe nuclei) were analyzed by PLS correlation with adjustment variables for age, sex, and disease duration. The relationship between tau PET and MRI volume within each tauopathy subgroup (AD, CBS, PSP-RS, and PSP-nonRS) was evaluated by testing the largest singular value with a non-parametric bootstrap method. For subgroups with a significant tau PET-MRI volume relationship, we extracted the set of VOIs with significant salience for the tau SUVR (i.e., *Z*-statistic > 2) and assessed the partial correlation between the tau SUVR of these VOIs and total GM volume ratio with bootstrapping. The relationships between the regional or global SUVRs of tau PET findings and clinical motor and cognitive symptoms severity were analyzed by Pearson correlation adjusted for age. The relationships between the regional or global SUVRs of tau PET findings and plasma biomarkers were leveraged by Pearson correlations adjusted for age. We used the area under the receiver operating characteristic (ROC) curve (AUC) to quantify the model's diagnostic performance for exploring the ability of ^18^F-Florzolotau PET and brain volumetric measurements to distinguish between patients with 4R tauopathy parkinsonism syndrome and other groups. Statistical analyses were performed in MedCalc version 20.109 (MedCalc Software Ltd, Ostend, Belgium), and a PLS correlation was performed in R version 4.3.1 (https://www.R-project.org/). A *p-*value < 0.05 was considered significant, and a 95% confidence interval (CI) is given when appropriate.

## Results

### Clinical characteristics

The clinical characteristics of the 80 enrolled participants are summarized in Table 1. Compared to the control group, patients in the individual disease groups were older, especially those in the PSP-RS group, and had a worse cognitive status. The proportion of men was higher in the PSP-RS and PSP-nonRS groups than other groups.

**Table 1.** Clinical characteristics of participants in the current study.

	Control (n = 18)	PD (n* = 8)*	*CBS (n = 9)*	*PSP-nonRS (n = 24)*	*PSP-RS (n = 11)*	*AD (n = 10)*	*p*
**Clinical Characteristics**							
Age (y)	63.8 ± 10.6	70.0 ± 5.7	65.6 ± 9.7	67.4 ± 7.6	73.1 ± 9.1	66.0 ± 8.1	0.12
Sex (male, n, %)	7 (38.9)	3 (37.5)	3 (33.3)	17 (70.8)	8 (72.7)	3 (30.0)	0.07
Disease Duration (y)	N.A.	5.8 ± 4.6	5.2 ± 2.0	5.2 ± 4.5	4.7 ± 3.0	3.7 ± 1.9	0.39
UPDRS part III score	N.A.	28.1 ± 6.5	35.0 ± 13.4	N.A.	N.A.	N.A.	-
PSP rating scale score	N.A.	N.A.	N.A.	33.4 ± 14.6	45.1 ± 17.2	N.A.	-
LEDD (mg)	N.A.	819 ± 383	658 ± 247	649 ± 293	745 ± 328	N.A.	-
MMSE	29.0 ± 1.1	27.3 ± 2.8	21.7 ± 8.0	25.2 ± 4.3	20.6 ± 8.2	19.3 ± 5.8	<0.01**
MoCA	28.0 ± 1.4	26.0 ± 3.6	18.0 ± 8.5	23.3 ± 5.4	18.0 ± 7.4	16.7 ± 6.3	<0.01**
**Plasma Biomarkers**							
Total tau (pg/ml)	3.2 ± 1.2	3.3 ± 0.9	5.3 ± 2.1	5.2 ± 2.3	4.3 ± 1.8	4.6 ± 2.3	0.03*
pTau181 (pg/ml)	1.3 ± 0.6	1.5 ± 0.7	3.5 ± 1.4	3.3 ± 1.5	2.8 ± 0.9	3.6 ± 2.2	<0.01**
NfL (pg/ml)	11.1 ± 9.0	29.3 ± 13.8	54.1 ± 17.6	40.7 ± 22.8	46.4 ± 20.3	30.4 ± 15.8	<0.01**
GFAP (pg/ml)	91.7 ± 57.3	151.0 ± 93.8	190.3 ± 89.1	195.8 ± 88.2	174.8 ± 82.3	268.4 ± 112.7	0.02*

Data are presented as mean ± S.D; baseline characteristics are compared by ANOVA or chi-squared test. MMSE: Mini-Mental Status Examination; MoCA: Montreal Cognitive Assessment; NfL: neurofilament light chain; GFAP: glial fibrillary acidic protein; PD: Parkinson's disease; CBS: corticobasal syndrome; PSP-RS: progressive supranuclear palsy-Richardson syndrome; PSP-nonRS: progressive supranuclear palsy-non-Richardson syndrome; AD: Alzheimer's disease. N.A.: not available; *p < 0.05, **p < 0.01.

### ^18^F-Florzolotau PET distribution patterns

**Figure 1.**
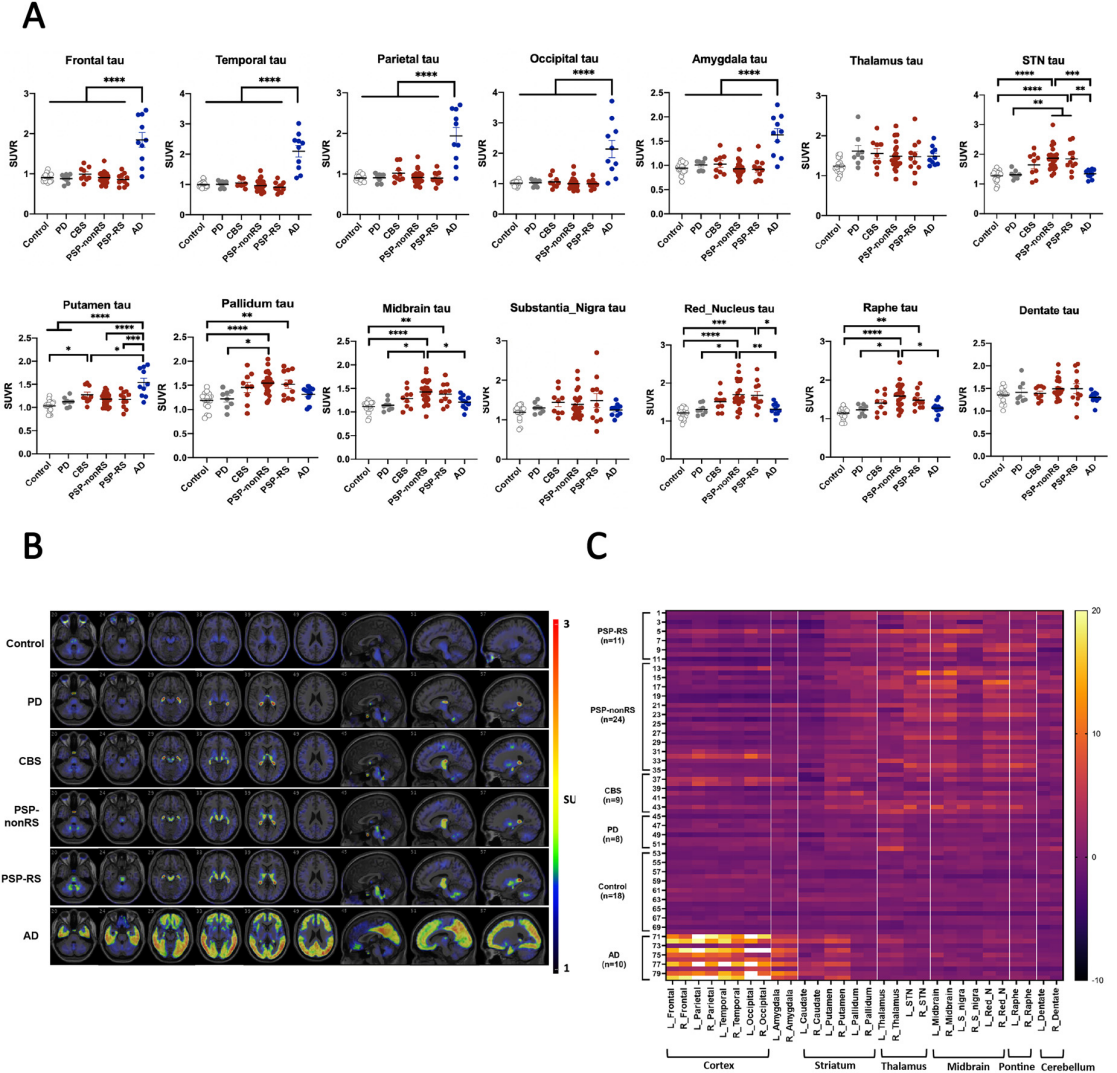
**Regional SUVRs and representative ^18^F-florzolotau PET images in participants from individual groups.** (A) Comparison of regional ^18^F-florzolotau SUVRs between groups adjusted for age and sex. (B) Representative cases of ^18^F-florzolotau PET imaging from each group showing different distribution patterns. The intensity of tracer uptake is shown as the SUVR with cerebellar crus as the reference. (C) Heat map of regional ^8^F-florzolotau uptake patterns for each VOI in each subject. Data are presented based on the individual SUVR Z-scores among each VOI. *p < 0.05, **p < 0.01, ***p < 0.001, and ****p < 0.0001 by Bonferroni corrected post hoc pairwise comparison. STN: subthalamic nucleus; L: left; R: right.

Compared to controls or other disease groups, patients with PSP and CBS had significantly higher ^18^F-florzolotau uptake in subcortical regions of the STN, pallidum, midbrain, red and raphe nucleus, especially those with PSP (Figure 1A, Supplementary Table 1). However, among PSP subtypes, there was no regional difference in tau uptake between PSP-RS and PSP-nonRS. For patients with CBS, tau tracer binding tended to be higher in the frontal and parietal cortex, though it did not reach significant levels, whereas the tau tracer retention in the putamen was remarkably higher than in controls (*p *= 0.01, Supplementary Table 1). In contrast, the cortical retention of ^18^F-florzolotau in the frontal, temporal, parietal, occipital, and amygdala regions was more increased in patients with AD than controls and other disease groups (all *p *< 0.0001).

Representative images of ^18^F-florzolotau binding in individual regions are shown in Figure 1B. Upon visual inspection, patients with PSP-RS, PSP-nonRS, and CBS had apparent tracer retention in subcortical and brainstem regions, whereas widespread tau tracer retention was obviously seen in lobar cortical regions in patients with AD. The tau tracer signals in these regions were relatively absent in patients with PD and controls. To illustrate the specific regional tau uptake patterns, heat maps were created based on the individual ^18^F-florzolotau SUVR Z-scores for each VOI from each subject (Figure 1C). Patients with PSP and CBS, the 4R tauopathy parkinsonism syndrome, were characterized by high ^18^F-florzolotau signals in the brainstem and subcortical nuclei, which were not observed in patients with PD, AD, or controls. Some of the patients with PSP or CBS also had higher cortical tau retention than controls or patients with PD.

### Correlations between ^18^F-florzolotau uptake and clinical severity

**Figure 2.**
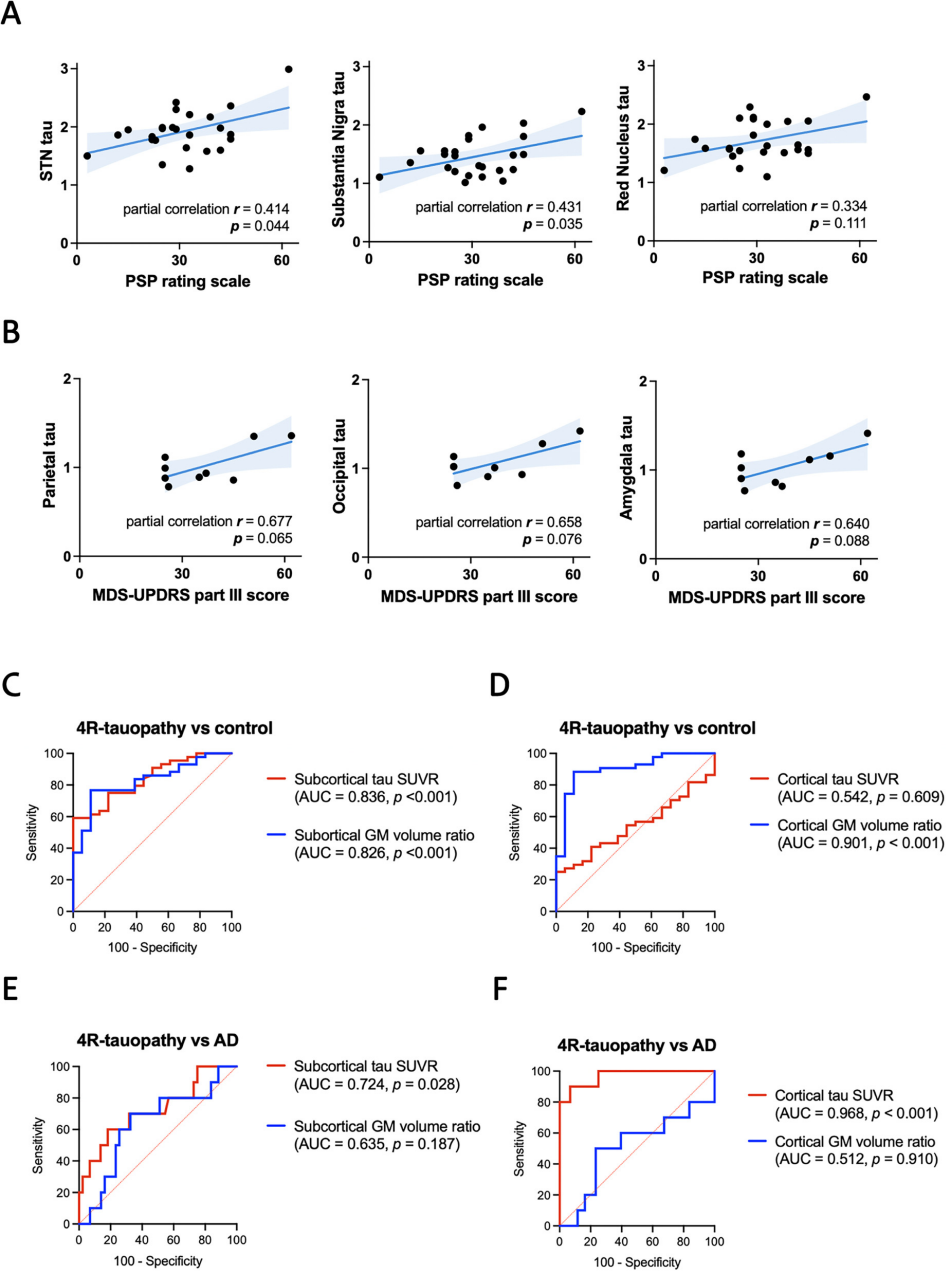
**Correlations between regional ^18^F-florzolotau uptake and clinical severity of PSP and CBS**. (A) Regional ^18^F-florzolotau uptake and PSP rating scores in patients with PSP. (B) Regional ^18^F-florzolotau uptake and MDS-UPDRS part III scores in patients with CBS.

As ^18^F-florzolotau uptake in the subcortical regions assists in differentiating PSP and CBS from controls and AD, we next examined the regional tau retention in individual subcortical regions and the clinical severity of PSP and CBS adjusted for age. Among patients with PSP, the PSP Rating Scale positively correlated with the amount of tau uptake in the STN (r = 0.414, *p *= 0.044), substantia nigra (r = 0.431, *p *= 0.035), and with nonsignificant trend found in red nucleus (r = 0.334, *p *= 0.111; Figure 2A). Among patients with CBS, the MDS-UPDRS part III motor scores are only marginally associated with tau retention in the parietal lobe (r = 0.677, *p *= 0.065), occipital lobe (r = 0.658, *p *= 0.076), and amygdala (r = 0.640, *p *= 0.088; Figure 2B). No association is observed between motor scores and tau retention in the subcortical regions.

### Associations between ^18^F-florzolotau uptake and structural brain volume

**Figure 3.**
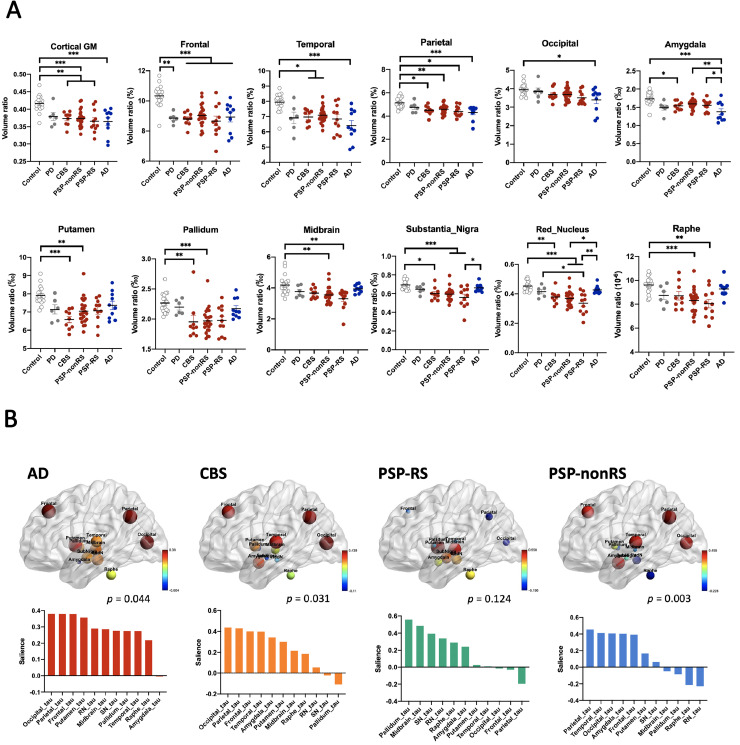
**Regional brain volume changes and association with ^18^F-florzolotau uptake in participants from individual groups.** (A) Comparison of total and regional volume ratios between groups adjusted for age. (B) Prediction of structural volume changes by regional ^18^F-florzolotau uptake in each tauopathy subgroup (AD, CBS, PSP-RS, PSP-nonRS). The bar charts below indicate saliences for tau SUVRs in each VOI. Salience with a larger absolute value implies greater predictive power for structural volume changes. *p < 0.05, **p < 0.01, and ***p < 0.001 by Bonferroni corrected post hoc pairwise comparison of the relationship between tau PET and volume parameters. GM: gray matter; RN: red nucleus; SN: substantia nigra; STN: subthalamic nucleus.

The cortical and subcortical structural volume ratios on brain MRI adjusted for age and sex are illustrated in Figure 3A for controls and different disease groups. Control participants had significantly thicker cortical volumes than the tauopathy groups (all *p *< 0.01), especially in the frontal lobe region (*p *< 0.001 vs. all tauopathy groups, Supplementary Table 2). On the other hand, patients with 4R tauopathy parkinsonism syndrome had remarkably smaller subcortical volumes than controls (Figure 3A). Compared to control participants, patients with PSP had significantly reduced volumes in the midbrain, substantia nigra, red nucleus, raphe nucleus, putamen, and pallidum (all *p *< 0.001, Supplementary Table 2). Compared to AD, patients with PSP had significantly smaller volumes in the red nucleus (*p *= 0.0017 for PSP-RS, *p *= 0.024 for PSP-nonRS) and substantia nigra (*p *= 0.042 for PSP-RS). In addition, patients with CBS had smaller subcortical volumes than controls in the putamen, pallidum, red nucleus, and substantia nigra (all *p *< 0.01, Supplementary Table 2).

Next, we examined whether a correlation exists between ^18^F-florzolotau uptake and regional brain volume changes in both the cortical and subcortical regions. The SUVRs of tau tracer ^18^F-florzolotau and structural volume ratios regarding multiple VOIs were analyzed in PLS correlation models. Tau tracer retention negatively correlated with structural volume changes in individuals with AD (partial correlation *r *= −0.451, *p *= 0.044), CBS (*r *= −0.484, *p *= 0.031), or PSP-nonRS (*r *= 0.469, *p *= 0.003), but not those with PSP-RS (*p *= 0.124; Figure 3B, details in Supplementary Table 3). For the AD group, regional tau SUVRs in the frontal, temporal, parietal, putamen, substantia nigra, and raphe nucleus regions had significant salience, with a remarkably correlation with the GM volume ratio. For the CBS group, significant tau SUVRs in VOIs, including the frontal, temporal, parietal, occipital, amygdala, putamen, midbrain, and raphe nucleus regions, with partial correlation with the GM volume ratio. For the PSP-nonRS group, only the frontal, temporal, parietal, occipital, and amygdala regions had significant saliences. Although a significant PLS correlation was not found in patients with PSP-RS, the regions with higher saliences were the pallidum, midbrain, substantia nigra, and red nucleus, a pattern that was different from the PSP-nonRS group.

### Diagnostic performance of ^18^F-florzolotau PET and brain MRI for 4R tauopathy parkinsonism

We then compared the diagnostic performance of 18F-florzolotau PET and brain MRI in distinguishing 4R tauopathy parkinsonism from both controls and AD. Further ROC curve analyses revealed that subcortical ^18^F-florzolotau uptake satisfactorily differentiated 4R tauopathy parkinsonism syndrome from controls (area under curve (AUC) 0.836, *p *< 0.001, Figure 2C), but not cortical tau uptake (AUC 0.542, *p *= 0.609, Figure 2D). On the other hand, while compared to patients with AD, ^18^F-APN-1607 tau PET SUVR shows higher values in patients with 4R tauopathy parkinsonism in subcortical regions (AUC 0.724, *p *= 0.028; Figure 2E) and lower values in cortical regions (AUC 0.968, *p *< 0.001; Figure 2F). In regard to the structural volume measurements, although the diagnostic performance of GM volumes is satisfactory in differentiating 4R tauopathy parkinsonism from controls in both cortical and subcortical regions (both *p *< 0.001) (Figure 2C, D), GM volumes are not as effective in distinguishing 4R tauopathy parkinsonism from AD in either cortical (AUC 0.512, *p *= 0.910) or subcortical (AUC 0.635, *p *= 0.187) regions (Figure 2E, F). This suggests that ^18^F-APN-1607 tau PET provides a higher diagnostic performance for differentiating 4R tauopathy parkinsonism from AD compared to brain volumetric measurements.

### Associations between ^18^F-florzolotau uptake and plasma biomarkers

**Figure 4.**
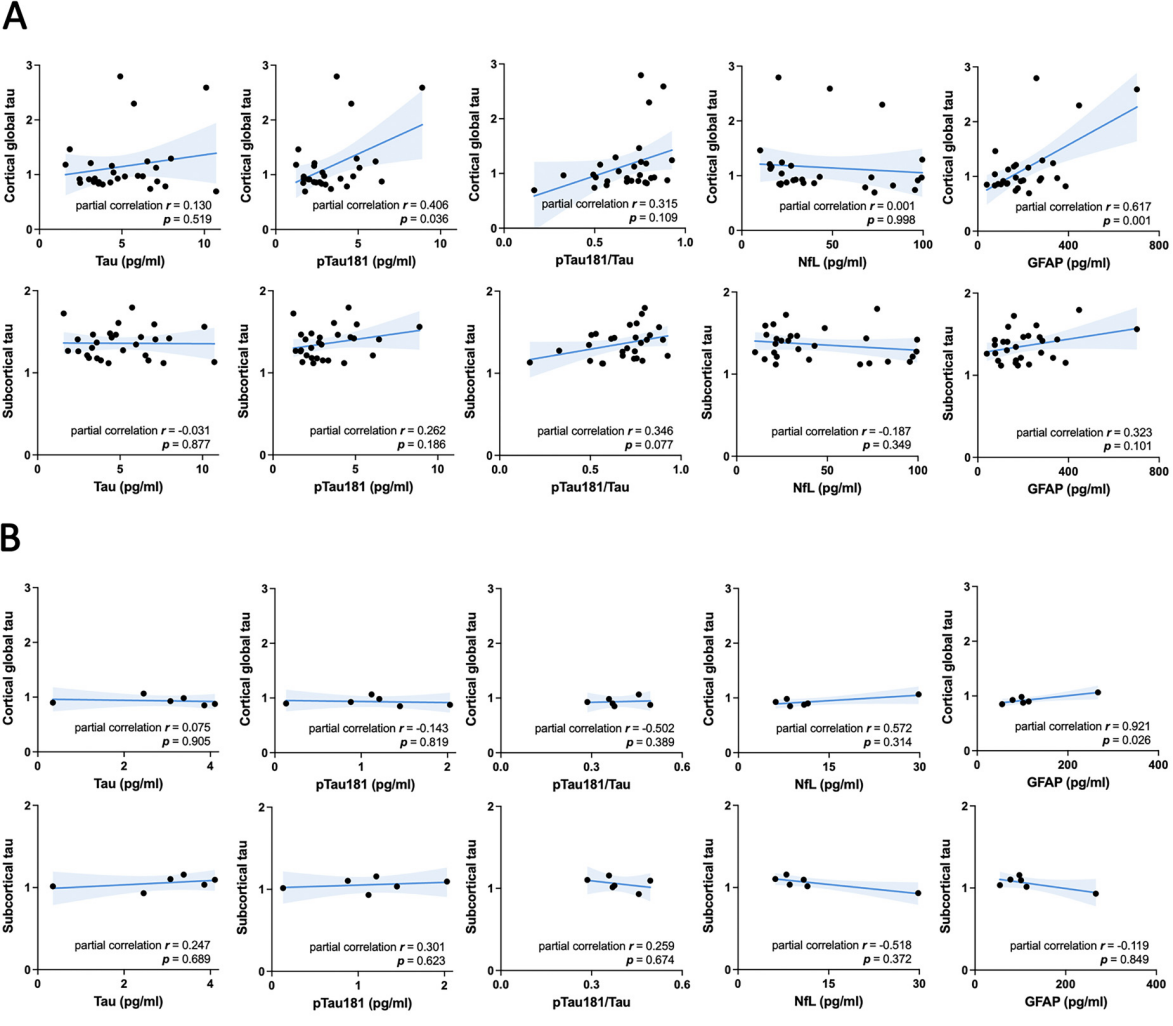
**Correlations between plasma biomarkers and cortical or subcortical ^18^F-florzolotau burden.** (A, B) Correlation of plasma biomarker and ^18^F-florzolotau uptake among patients with tauopathy disorders, including PSP-RS, PSP-nonRS, CBS, and AD, (A) and control participants (B).

As the plasma level of phosphorylated tau was recently adopted as a surrogate marker of tau retention in the living brains of patients with tauopathy,^25^ we explored the correlation between ^18^F-florzolotau uptake and plasma biomarkers. Compared to controls and patients with PD, the plasma levels of total tau and pTau181 were higher in patients with tauopathy disorders, including PSP, CBS, and AD (Table 1). In addition to tau, the plasma levels of neurodegeneration marker NfL and neuroinflammation marker GFAP were elevated in all disease groups compared to controls (*p *< 0.01, Table 1). For the tauopathy groups—comprising PSP-RS, PSP-nonRS, CBS, and AD—while controlling for age, we observed significant positive correlations between cortical tau retention and plasma concentrations of GFAP (*r *= 0.617, *p *= 0.001) and pTau181 (*r *= 0.406, *p *= 0.036; Figure 4A). In contrast, no associations were found between cortical or subcortical tau retention and plasma biomarkers in the control participants (Figure 4B).

## Discussion

In the current study, we demonstrated that the topographic distribution of ^18^F-florzolotau uptake can distinguish patients with 4R tauopathy parkinsonism (PSP and CBS) from controls, PD, and AD. The ^18^F-florzolotau binding was higher in subcortical regions, especially the STN, pallidum, midbrain, red nucleus, and raphe nucleus, in patients with PSP, whereas the ^18^F-florzolotau uptake in patients with CBS was significantly increased in the putamen and tended to be higher in the frontal and parietal regions. Subcortical ^18^F-florzolotau tau retention could differentiate patients with 4R tauopathy parkinsonism from controls. In addition, tau retention in both cortical and subcortical regions serves as a superior marker for differentiating 4R tauopathy parkinsonism from AD compared to brain volumetric measurements. The motor severity of PSP positively correlated with tau burden in STN and substantia nigra. Furthermore, regional tau burden was associated with MRI volume changes, whereas the global cortical tau burden positively correlated with plasma levels of GFAP and pTau181 in the tauopathy patient groups, including 4R tauopathy parkinsonism and AD.

Recently, the second-generation PET tau tracer^18^F-florzolotau was shown to capture diverse tau deposits in postmortem brains from patients with PSP and CBS.^26^ Subsequent assays demonstrated that ^18^F-florzolotau has a stronger binding affinity for 4R tau fibrils compared to the first-generation tau tracer.^27^ Further investigations revealed that ^18^F-florzolotau PET can assist in differentiating PSP and CBS from control participants based on the topographical distribution of tau tracer in the living brains of patients.^28–30^ Consistently, we found that ^18^F-florzolotau uptake is increased in the STN, pallidum, midbrain, red nucleus, and raphe nucleus in patients with PSP compared to controls and other disease groups. We also observed that the tau burden in the STN correlates with the clinical severity in PSP. Our findings not only validate previous studies^28–30^ to support the promising role of ^18^F-florzolotau PET imaging in distinguishing PSP from controls and PD, but are also in line with neuropathology findings that tau load starts from the pallido-nigro-luysian axis, with the most abundant tau depositions observed in the pallidum, STN, and substantia nigra in PSP.^31^ In addition, our study did not find a different ^18^F-florzolotau binding pattern between the PSP-RS and PSP-nonRS subtypes, which is in accordance with previous studies that demonstrated comparable tau distribution patterns of PSP-RS and other variants of PSP.^28–30^ These clinical observations also reflect the neuropathology findings of a pathological tau burden with uniform distribution pattern in PSP regardless of different clinical subtype.^31^ In addition to PSP, we observed that the tau tracer binding was most obvious in the putamen and tended to increase in the frontal and parietal cortex in patients with CBS. Our results are consistent with recent observations of an increased ^18^F-florzolotau signal in the frontal and parietal cortex, encompassing the precentral gyrus, postcentral gyrus, and supplementary motor areas in patients with CBS.^11^ We also observed that the putamen is among the areas with the highest tau burden in patients with CBS. Thus, subcortical tau tracer retention could satisfactorily assist in differentiating 4R tauopathy parkinsonism syndrome (i.e., PSP and CBS) from controls and AD. However, as CBS is a pathologically heterogenous disorder, further longitudinal and clinicopathological validation studies are needed to validate the application of ^18^F-florzolotau signal in cortical regions as an imaging hallmark of CBS.

In this study, clinical motor severity is associated with PSP tau burden in STN and substantia nigra. These findings indicate that the motor severity is related to tau-accumulation in STN in the indirect pathway of basal ganglia, as well as substantia nigra within the dopaminergic neurotransmitter system. The results are in accordance with a previous ^18^F-florzolotau tau PET study in patients with PSP.^10^ However, the correlations between clinical motor scores and tau burden in sub-cortical brain regions are not significant in CBS, which maybe limited by the small sample size. The current study also revealed regional 18F-florzolotau uptake associated with structural volume changes among patients with PSP, CBS, and AD. After adjusting for age and sex, significant regional tau uptake was observed in patients with CBS, PSP-nonRS, and AD, correlating with GM cortical volume changes in the frontal, temporal, parietal, and occipital regions. In contrast, no association between 18F-florzolotau uptake and volume change was found in patients with PSP-RS in this model. The lack of statistical significance in the PSP-RS group is likely due to the limited sample size compared to the PSP-nonRS group. Although the overall statistical significance was not reached for tau burden and brain atrophy in PSP-RS, the regions with higher salience, such as subcortical areas including the pallidum, midbrain, substantia nigra, and red nucleus, differ significantly from those in the PSP-nonRS group. These findings may offer insights into the interaction between tau burden and neurodegeneration in different tauopathy syndromes.^32^

In addition to structural brain volume changes, we found a significant correlation between the ^18^F-florzolotau tracer burden and plasma levels of GFAP, as well as a marginal correlation with plasma concentrations of pTau181. Although a recent study found that the pTau181 level is reduced in patients with PSP compared to controls,^33^ we found that plasma levels of total tau and pTau181 were increased in patients with PSP or CBS compared to controls or patients with PD. However, the correlation between plasma levels of pTau181 and the ^18^F-florzolotau burden in brains is only marginal. Plasma pTau181 has been strongly correlated with tau PET signals across the cortex in patients with AD.^25,34^ Based on the different structures and isoforms of tau in AD and PSP, plasma pTau181 may not reflect tau burden in patients with 4R tauopathy parkinsonism. Furthermore, plasma levels of phosphorylated tau measured by currently available assays may be influenced by brain amyloid^35^ and, therefore, could not accurately reflect brain tau deposits. A novel immunoassay was recently developed to quantify N- and C-terminally truncated p-tau fragments (mid-p-tau181) in human plasma.^36^ The mid-p-tau181 assay had stronger correlations with tau PET accumulation than the conventional assay in patients with AD and accurately distinguished between tau PET-positive and -negative cases.^36^ The methods for blood-based tau measurement and specific substrates used for detecting plasma tau are crucial for developing plasma biomarkers and a correlation with tau PET imaging findings. We also observed that higher plasma GFAP levels correlated with increased binding of ^18^F-florzolotau in the cortex, especially in broad regions of the frontal, parietal, and temporal lobes in patients with tauopathy disorders. Consistent with our findings, one recent study showed that plasma GFAP correlated with tau tracer uptake across the cortex in patients with PSP.^33^ Furthermore, higher GFAP levels were reported in CSF and blood samples from individuals with AD and other dementias compared to healthy controls.^37^ GFAP is an astrocytic cytoskeletal protein expressed mainly in the astrocytes of the brain, and serves as a marker of abnormal activation and proliferation of astrocytes, known as astrogliosis.^38^ Notably, recent neuropathological evidence indicated that astrogliosis is one of the main pathological features in PSP.^31^ Though neuronal tau accumulation is central to the pathogenesis of PSP, astroglial tau accumulation combined with astrogliosis may precede neuronal tau pathology in the striatum, cortical regions, globus pallidus, and cerebellar white matter.^31^ In addition, astrocytosis and tau accumulation within astrocytes as astrocytic plaques are most abundant in the prefrontal and premotor areas of the cerebral cortex and basal ganglia, including the caudate and putamen, of post-mortem brains from individuals with CBS.^39,40^ Our findings combined with previous observations suggest that blood levels of GFAP may reflect astrocytosis in the brain and could serve as a surrogate marker of PSP and CBS.

The major strength of the current study is that we adopted multimodal tau-directed imaging and biofluid markers in the differential diagnosis of 4R tauopathy parkinsonism syndrome. Another strength is that we assessed the relationship between the burden of tau tracer ^18^F-florzolotau and brain volume atrophy or plasma biomarker changes. However, this study has several limitations. First, the relatively small sample size in the PSP-RS and CBS groups limited our ability to distinguish PSP-RS from other subtypes of PSP. Second, the diagnoses of enrolled participants were established based on clinical criteria rather than postmortem neuropathological confirmation. The lack of amyloid PET imaging scans for the assessment of co-morbid amyloid neuropathology may lead to the misclassification of enrolled patients. However, all tau PET scans were reviewed by nuclear imaging experts, which supported the clinical diagnosis; for example, none of the patients with CBS exhibited typical tau accumulation in AD-specific temporoparietal areas. Third, the control participants in our study are younger than those with the PSP-RS subtype. To account for potential confounding biases, we adjusted for age in all statistical analyses. Further longitudinal studies incorporating larger samples of patients with 4R tauopathy disorders and comprehensively integrated markers, including blood-based biomarkers related to amyloid, α-synuclein, and different phosphorylated tau proteins, and seeding amplification assays for tau in combination with tau PET imaging are needed for future tau-targeting therapy in patients with 4R tauopathy parkinsonism syndrome.

## Supplemental Material

sj-docx-1-pkn-10.1177_1877718X241298192 - Supplemental material for Subcortical tau burden correlates with regional brain atrophy and plasma markers in four-repeat tauopathy parkinsonism Supplemental material, sj-docx-1-pkn-10.1177_1877718X241298192 for Subcortical tau burden correlates with regional brain atrophy and plasma markers in four-repeat tauopathy parkinsonism by Cheng-Hsuan Li, Sung-Pin Fan, Ming-Chieh Shih, Yi-Hsin Weng, Ta-Fu Chen, Hsun Li, Mei-Fang Cheng, Ming-Che Kuo, Pei-Ling Peng, Makoto Higuchi, Ing-Tsung Hsiao, Kun-Ju Lin and Chin-Hsien Lin in Journal of Parkinson's Disease
