# Supplementary material for: Subcortical tau burden correlates with regional brain atrophy and plasma markers in four-repeat tauopathy parkinsonism
Source: J Parkinsons Dis. 2024 Dec 8:1877718X241298192. Online ahead of print. doi: 10.1177/1877718X241298192 (PMC13347418; doi:10.1177/1877718X241298192)
Supplement: Supplementary file 1 [file sj-docx-1-pkn-10.1177_1877718X241298192.docx]

**Supplemental Material**

**Subcortical tau burden correlates with regional brain atrophy and plasma markers in four-repeat tauopathy parkinsonism**

**Supplemental Table 1.** Comparison of regional SUVRs of ^18^F-florzolotau PET imaging between participants of different groups in the current study.

| **Regions** | **Control**  **(n=18)** | **PD**  **(n=8)** | **CBS**  **(n=9)** | **PSP-nonRS**  **(n=24)** | **PSP-RS**  **(n=11)** | **AD**  **(n=10)** | **Comparison (Bonferroni corrected *p<*0.05)** |
| --- | --- | --- | --- | --- | --- | --- | --- |
| **Frontal** | 0.903 | 0.878 | 0.994 | 0.905 | 0.856 | 1.845 | AD>control, PD, CBS, PSP-nonRS, PSP-RS (all *p*<0.0001) |
| **Temporal** | 0.996 | 1.006 | 1.048 | 0.963 | 0.909 | 2.099 | AD>control, PD, CBS, PSP-nonRS, PSP-RS (all *p*<0.0001) |
| **Parietal** | 0.902 | 0.905 | 1.018 | 0.913 | 0.898 | 1.939 | AD>control, PD, CBS, PSP-nonRS, PSP-RS (all *p*<0.0001) |
| **Occipital** | 1.017 | 1.031 | 1.059 | 0.998 | 0.997 | 2.137 | AD>control, PD, CBS, PSP-nonRS, PSP-RS (all *p*<0.0001) |
| **Amygdala** | 0.942 | 1.007 | 1.027 | 0.927 | 0.918 | 1.627 | AD>control, PD, CBS, PSP-nonRS, PSP-RS (all *p*<0.0001) |
| **Thalamus** | 1.238 | 1.617 | 1.555 | 1.489 | 1.472 | 1.489 |  |
| **STN** | 1.275 | 1.314 | 1.643 | 1.870 | 1.845 | 1.340 | PSP-nonRS>control (p<0.0001); PSP-nonRS>PD (*p*=0.0018); PSP-nonRS>AD (p=0.0006); PSP-RS>control (*p*=0.0001); PSP-RS>AD (*p*=0.0047); PSP-RS>PD (*p*=0.0069) |
| **Putamen** | 1.035 | 1.125 | 1.273 | 1.182 | 1.174 | 1.538 | AD>control, PD, PSP-nonRS (*p*<0.0001); AD>PSP-RS (*p*=0.0004); AD>CBS (*p*=0.018); CBS>control (*p*=0.0169) |
| **Pallidum** | 1.187 | 1.221 | 1.457 | 1.549 | 1.523 | 1.316 | PSP-nonRS>control (*p*=0.0001); PSP-RS>control (*p*=0.0033); PSP-nonRS>PD (*p*=0.0239) |
| **Midbrain** | 1.116 | 1.145 | 1.286 | 1.425 | 1.379 | 1.211 | PSP-nonRS>control (*p* <0.0001); PSP-nonRS>AD (*p*=0.0485); PSP-nonRS>PD (*p*=0.0145); PSP-RS>control (*p*=0.0017) |
| **Substantia nigra** | 1.192 | 1.299 | 1.439 | 1.389 | 1.484 | 1.246 |  |
| **Red nucleus** | 1.210 | 1.307 | 1.998 | 2.467 | 2.366 | 1.560 | PSP-nonRS>control (*p* <0.0001); PSP-nonRS>AD (*p*=0.0046); PSP-nonRS>PD (*p*=0.0166); PSP-RS>control (*p*=0.0003); PSP-RS>AD (*p*=0.0261) |
| **Raphe nucleus** | 1.142 | 1.227 | 1.400 | 1.584 | 1.480 | 1.274 | PSP-nonRS>control (*p* <0.0001); PSP-nonRS>AD (*p*=0.0149); PSP-nonRS>PD (*p*=0.0143); PSP-RS>control (*p*=0.0015) |
| **Dentate** | 1.356 | 1.412 | 1.391 | 1.494 | 1.495 | 1.303 |  |

Data presented in SUVR, using cerebellar crus as reference. STN: subthalamic nucleus.

**Supplementary Table 2.** Linear regression analysis between regional ^18^F-florzolotau uptake and cognitive function adjusted for age and sex.

| **Regions** | **PSP** | | | | | | **CBS** | | | | | |
| --- | --- | --- | --- | --- | --- | --- | --- | --- | --- | --- | --- | --- |
|  | with MMSE | | with MoCA | | | with MMSE | | | with MoCA | | |  |
|  | β [95% CI] | *p* | | β [95% CI] | *p* | | β [95% CI] | *p* | | β [95% CI] | *p* | |
| **Frontal** | 1.76 [-13.9, 17.4] | 0.82 | | 4.84 [-11.8, 21.5] | 0.59 | | -8.71 [-60.9, 43.5] | 0.69 | | -3.98 [-60.4, 52.4] | 0.86 | |
| **Temporal** | 4.59 [-7.84, 17.0] | 0.46 | | 9.16 [-3.89, 22.2] | 0.16 | | -18.4 [-84.6, 47.9] | 0.51 | | -15.9 [-87.7, 55.9] | 0.59 | |
| **Parietal** | 6.82 [-10.3, 17.6] | 0.60 | | 6.83 [-7.99, 21.7] | 0.35 | | -12.5 [-53.7, 28.7] | 0.47 | | -8.86 [-54.2, 36.5] | 0.64 | |
| **Occipital** | 5.54 [-7.49, 18.6] | 0.39 | | 9.53 [-4.25, 23.3] | 0.17 | | -10.1 [-59.5, 39.4] | 0.62 | | -5.14 [-58.9, 48.6] | 0.82 | |
| **Amygdala** | 6.61 [-6.17, 19.4] | 0.30 | | 8.46 [-5.19, 22.1] | 0.22 | | -7.62 [-54.9, 39.7] | 0.70 | | -5.63 [-56.5, 45.2] | 0.79 | |
| **Thalamus** | 0.79 [-5.27, 6.85] | 0.79 | | 2.16 [-4.37, 8.69] | 0.50 | | 10.2 [-13.0, 33.5] | 0.31 | | 8.25 [-17.8, 34.3] | 0.45 | |
| **STN** | 0.79 [-5.24, 6.81] | 0.79 | | -1.46 [-7.87, 4.95] | 0.65 | | 10.4 [-11.0, 31.8] | 0.27 | | 10.4 [-13.4, 33.5] | 0.32 | |
| **Putamen** | 1.50 [-13.5, 16.5] | 0.84 | | 0.97 [-15.2. 17.2] | 0.90 | | 6.52 [-43.2, 56.3] | 0.34 | | 9.46 [-43.0, 61.9] | 0.66 | |
| **Pallidum** | 8.19 [-0.90, 17.3] | 0.08 | | 6.24 [-3.91, 16.4] | 0.22 | | 10.8 [-17.0, 38.6] | 0.36 | | 9.97 [-20.3, 40.3] | 0.44 | |
| **Midbrain** | 3.59 [-7.36, 14.5] | 0.51 | | 3.38 [-8.77, 15.5] | 0.57 | | 12.6 [-35.5, 60.7] | 0.53 | | 13.5 [-37.7, 64.7] | 0.53 | |
| **Substantia nigra** | 2.16 [-3.07, 7.39] | 0.41 | | 0.87 [-4.80, 6.54] | 0.76 | | 15.7 [-12.5, 43.8] | 0.21 | | 13.7 [-18.2, 45.6] | 0.32 | |
| **Red nucleus** | 1.10 [-5.66, 7.87] | 0.74 | | -1.21 [-8.53, 6.11] | 0.74 | | 18.7 [-15.7, 53.0] | 0.22 | | 17.6 [-20.5, 55.6] | 0.29 | |
| **Raphe** | 0.51 [-8.44, 9.47] | 0.91 | | 0.08 [-9.52, 9.67] | 0.99 | | 11.6 [-21.9, 45.1] | 0.41 | | 12.4 [-23.3, 48.1] | 0.41 | |
| **Dentate** | -0.23 [-9.37, 8.91] | 0.96 | | -3.31 [-13.0, 6.36] | 0.49 | | -2.39 [-87.0, 82.3] | 0.95 | | -0.01 [-0.01, 0.01] | 0.68 | |

β, coefficient of regional tau SUVR; CI: confidence interval; STN: subthalamic nucleus.

**Supplementary Table 3.** Cortical, subcortical and regional structural brain volume ratios of participants in different groups.

| **Regions** | **Control**  **(n=18)** | **PD**  **(n=8)** | **CBS**  **(n=9)** | **PSP-nonRS**  **(n=24)** | **PSP-RS**  **(n=11)** | **AD**  **(n=10)** | **Comparison (Bonferroni corrected *p*<0.05)** |
| --- | --- | --- | --- | --- | --- | --- | --- |
| Cortical GM (%) | 41.6 | 37.9 | 37.3 | 37.4 | 36.6 | 36.5 | AD<control (*p*=0.0003); PD<control (*p*=0.0038); CBS<control (*p*=0.0001); PSP-nonRS<control (*p*=0.0001); PSP-RS<control (*p*=0.0002) |
| Subcortical GM (%) | 4.19 | 3.49 | 3.29 | 3.47 | 3.21 | 3.50 | CBS<control (*p*=0.0077); PSP-nonRS<control (*p*=0.029); PSP-RS<control (*p*=0.0137) |
| Frontal (%) | 10.33 | 8.84 | 8.79 | 9.02 | 8.66 | 8.92 | AD<control (*p*=0.0002); PD<control (*p*=0.0034); CBS<control (*p*=0.0001); PSP-nonRS<control (*p*<0.0001); PSP-RS<control (*p*=0.0001) |
| Temporal (%) | 7.94 | 6.91 | 6.97 | 7.09 | 6.85 | 6.41 | AD<control (*p*<0.0001); CBS<control (*p*=0.0151); PSP-nonRS<control (*p*=0.0103) |
| Parietal (%) | 5.13 | 4.75 | 4.50 | 4.57 | 4.40 | 4.30 | AD<control (*p*=0.0002); CBS<control; PSP-nonRS<control (*p*=0.0058); PSP-RS<control (*p*=0.0157) |
| Occipital (%) | 3.94 | 3.85 | 3.68 | 3.68 | 3.51 | 3.40 | AD<control (*p*=0.0127) |
| Amygdala (‰) | 1.74 | 1.49 | 1.54 | 1.59 | 1.55 | 1.38 | AD<control (*p*<0.0001); AD<PSP-nonRS (*p*=0.0026); AD<PSP-RS (*p*=0.0153); CBS<control (*p*=0.0492) |
| Putamen (‰) | 7.91 | 7.14 | 6.59 | 7.04 | 7.07 | 7.37 | CBS<control (*p*<0.0001); PSP-nonRS<control (*p*=0.0013) |
| Pallidum (‰) | 2.27 | 2.20 | 2.17 | 1.97 | 1.98 | 2.17 | CBS<control (*p*=0.0098); PSP-nonRS<control (*p*=0.0009) |
| Midbrain (‰) | 4.18 | 3.76 | 3.66 | 3.55 | 3.31 | 3.94 | PSP-nonRS<control (*p*=0.0052); PSP-RS<control (*p*=0.0036) |
| Substantia nigra (‰) | 0.69 | 0.65 | 0.60 | 0.59 | 0.56 | 0.66 | CBS<control (*p*=0.0249); PSP-nonRS<control (*p*=0.0006); PSP-RS<control (*p*=0.0005); PSP-RS<AD (*p*=0.0415) |
| Red nucleus (‰) | 0.45 | 0.42 | 0.38 | 0.37 | 0.34 | 0.43 | PSP-nonRS<control (*p*<0.0001); PSP-nonRS<AD (*p*=0.0238); PSP-RS<control (*p*<0.0001); PSP-RS<AD (*p*=0.0017); PSP-RS<PD (*p*=0.0354); CBS<control (*p*=0.0053) |
| Raphe nucleus (10^-6^) | 9.62 | 8.75 | 8.71 | 8.30 | 8.04 | 9.28 | PSP-nonRS<control (*p*=0.0004); PSP-RS<control (*p*=0.0012) |

GM: gray matter

**Supplementary Table 4.** Correlations between ^18^F-Florzolotau uptake and structural brain volume in different disease groups.

| Group | VOIs with significant tau SUVR salience | Xvec (coefficient) | Partial correlation (95% CI) |
| --- | --- | --- | --- |
| AD | Frontal  Temporal  Parietal  Putamen  Substantia Nigra  Raphe Nucleus | 0.536  0.211  0.576  0.502  0.218  0.192 | -0.451 (-0.849 ~ -0.158) |
| CBS | Frontal  Temporal  Parietal  Occipital  Amygdala  Putamen  Midbrain  Raphe nucleus | 0.291  0.340  0.296  0.260  0.442  0.377  0.414  0.368 | -0.484 (-0.949 ~ -0.767) |
| PSP-nonRS | Frontal  Temporal  Parietal  Occipital  Amygdala | 0.391  0.434  0.518  0.502  0.371 | 0.469 (0.202 ~ 0.715) |

VOI: volumes of interest; SUVR: standard uptake value ratio; CI: confidence interval
